# Kinases Involved in Both Autophagy and Mitosis

**DOI:** 10.3390/ijms18091884

**Published:** 2017-08-31

**Authors:** Zhiyuan Li, Xin Zhang

**Affiliations:** High Magnetic Field Laboratory, Chinese Academy of Sciences, Hefei 230031, China

**Keywords:** kinases, autophagy, mitosis, cyclin-dependent kinases, Aurora, polo-like kinases

## Abstract

Both mitosis and autophagy are highly regulated dynamic cellular processes and involve various phosphorylation events catalysed by kinases, which play vital roles in almost all physiological and pathological conditions. Mitosis is a key event during the cell cycle, in which the cell divides into two daughter cells. Autophagy is a process in which the cell digests its own cellular contents. Although autophagy regulation has mainly been studied in asynchronous cells, increasing evidence indicates that autophagy is in fact tightly regulated in mitosis. Here in this review, we will discuss kinases that were originally identified to be involved in only one of either mitosis or autophagy, but were later found to participate in both processes, such as CDKs (cyclin-dependent kinases), Aurora kinases, PLK-1 (polo-like kinase 1), BUB1 (budding uninhibited by benzimidazoles 1), MAPKs (mitogen-activated protein kinases), mTORC1 (mechanistic target of rapamycin complex 1), AMPK (AMP-activated protein kinase), PI3K (phosphoinositide-3 kinase) and protein kinase B (AKT). By focusing on kinases involved in both autophagy and mitosis, we will get a more comprehensive understanding about the reciprocal regulation between the two key cellular events, which will also shed light on their related therapeutic investigations.

## 1. Introduction

Autophagy is an evolutionarily conserved process in which the cell digests its unwanted cellular contents to maintain homeostasis, meet nutrient and energy demand, as well as to defend pathogen infection or various stimuli. Autophagy related proteins—ATGs—are the major executor and organizer of autophagy machinery. Beclin-1/ATG6, the mammalian homolog of yeast Atg6, is the first ATG cloned in mammalian cells. ULK1 (Unc-51-like kinase 1), the mammalian homolog of yeast Atg1, is the only serine/threonine protein kinase in all known ATGs. Various signals induce the phagophore formation by recruiting ULK1 complex and class III PI 3-kinase (the mammalian homolog of yeast Vps34) complex. Sequentially, the Atg5–ATG12–ATG16l complex regulates the elongation step and then autophagy marker LC3/ATG8 induces the closure of double-membrane autophagosome. The autophagosome finally fuses with the lysosome to degrade its inner contents and recycle them to provide nutrients and energy [1,2].

Mitosis is a fast dynamic cellular process involving many dramatic changes such as nuclear envelope disassembly, mitotic spindle formation, Golgi breakdown, chromosome alignment and segregation as well as cell membrane remodeling [3,4]. It has been known that various mitotic kinases serve as the master of mitosis. Among them, the most important one is CDK1—the homolog of yeast Cdc2 (cell division cycle protein 2)—which functions with its type-A or B cyclin partners [5]. CDK1 controls the entry into mitosis from the G2 phase and manipulates the whole mitosis by phosphorylating several substrates, such as histone H1, cyclin B, vimentin and p60^c-src^ [6,7]. Furthermore, besides CDK1, other kinases such as Aurora, Polo-like kinase, Bub1/BubR1 and NEK (Never in mitosis gene A-related kinase) all play vital roles in mitotic progression.

Autophagy and mitosis are critical cellular events that have sophisticated and highly orchestrated temporal and spatial control systems. Although increasing evidence demonstrates that they are in fact intertwined, information about their internal links is still very limited and there are mixed opinions. It is interesting that some kinases, first thought to function only in mitosis or autophagy alone, were later proven to be involved in both processes [8,9]. In the following sections, we will summarize the known facts about such kinases, from their original functions discovered in one process to their later unraveled roles in the other.

## 2. Kinases Originally Involved in Mitotic Regulation Are Shown to Play Additional Roles in Autophagy

### 2.1. Cyclin-Dependent Kinases

Cyclin-dependent kinases (CDKs)—known as protein kinases—characterized by requiring a cyclin partner for its enzymatic activity, are first discovered for their roles in cell cycle regulation. CDKs play key roles in cell cycle and transcription regulation in response to intracellular and extracellular stimuli. In mammals, CDKs could be divided into three cell-cycle related subfamilies (CDK1, CDK4, CDK5) and five related transcription subfamilies (CDK7, CDK8, CDK9, CDK11, CDK20) [10]. Moreover, although most CDKs play roles in cell cycles, their functions could be compensated by other CDKs as revealed by genetic ablation, except for CDK1 [11].

However, emerging data showed that CDKs function in autophagy as well. Recently, Yuan’s group found that CDK1 and CDK5 phosphorylate VPS34 at T159 in their common substrate recognition motif to hinder the interaction between VPS34 and Beclin-1. Moreover, CDK5 also phosphorylates VPS34 at T668 to inhibit its lipid enzymatic activity, thus decreasing PI3P production and results in decreased autophagy in mitosis [12,13]. This finding revealed an underlying mechanism for autophagy regulation in mitosis.

In addition, mitotic kinase CDK11 was also implicated in autophagy regulation. CDK11 was first identified as a cell division control (CDC)-related kinase belonging to the p34^cdc2^ family [14]. The full-length CDK11^p110^ isoform contains an IRES (Internal ribosome entry site) and a caspase-3 site, which leads to the generation of a larger CDK11^p58^ and a smaller CDK11^p46^ isoform, respectively [15,16]. The CDK11^p58^ kinase isoform is generated in a mitosis-specific manner, suggesting that the isoform participates in mitotic regulation. Indeed, CDK11^p58^ is found to be required for centrosome maturation, bipolar spindle assembly, maintenance of sister chromatid cohesion and cytokinesis [17,18,19], duplication of centrioles [20], protection sister chromatid cohesion at centromeres in mitosis [21] and mitotic arrest [22]. Moreover, CDK11 functions in autophagy other than mitosis. For example, Wilkinson S et al. reported that Drosophila cyclin-dependent kinase PITSLRE, a human CDK11 ortholog, is a modulator of autophagy [23]. Loss of CDK11 at early time points appears to induce autophagy, but at later time points CDK11 is critically required for autophagic flux and cargo digestion. Since PITSLRE/CDK11 regulates autophagy in Drosophila and human cells, this kinase represents a novel conserved component of the autophagy machinery [23]. Since CDK11 has been reported to be perturbed in various cancers including osteosarcoma, liposarcoma, breast cancer, skin cancer, prostate cancer [24,25,26,27,28,29], investigation into the dual roles of CDK11 in mitosis and autophagy will shed light on the field of cancer therapy.

Other than the CDK1, CDK5 and CDK11 mentioned above, CDKL3 (cyclin-dependent kinase-like 3) was also found to be involved in autophagy regulation. CDKL3—a conserved CDC2-related kinase with features of both MAPKs and CDKs—is similar to human putative NKIAMRE which is lost in leukemic patients with chromosome 5q deletions [30,31]. Recently, a comprehensive siRNA screening indicates that CDKL3 knockdown decreased mTORC1 activity, suggesting that the autophagy stimulating activity of CDKL3 knockdown is mediated by mTORC1 inhibition [32]. Besides CDKs themselves, CDKIs (CDK inhibitors) p16, p19, and p21 induce autophagy in cancer associated fibroblasts and primary hepatocytes [33]. Moreover, p21 plays an essential role in determining the type of cell death, which positively regulates apoptosis-mediated cell death and negatively regulates autophagy-mediated cell death [34]. This suggests CDKs-related roles in autophagy regulation and autophagy associated cell death.

In the meantime, numerous questions still remain for the dual roles of CDKs in mitosis and autophagy. Do other CDKs participate in autophagy? Do the different isoforms of the same CDK, such as mitotic CDK11^p58^, function in autophagy regulation? Are there more mediators other than mTORC1 for autophagic functions of CDKs? Obviously, more investigations are necessary to solve these puzzles.

### 2.2. Aurora Kinases

Aurora kinases are serine/threonine kinases which include Aurora A, B and C. Aurora A and B are well studied but the role of Aurora C is relatively less explored. Aurora A is associated with centrosome maturation and separation, and thereby regulates spindle assembly and stability. Its activity peaks during G2–M transition. Aurora B, a chromosomal passenger protein, is a key player in chromosome segregation. In early mitosis, Aurora B concentrates at centromeres and kinetochores, the attachment sites of chromosomes to spindle microtubules. There, it ensures fidelity of cell division, including kinetochore stabilization, kinetochore–microtubule attachment, and the regulation of a surveillance mechanism named the spindle assembly checkpoint [35,36].

Other than its roles in mitosis, Aurora A kinase was recently found to be involved in autophagy as well. For example, the Fan group suggested that Curcumin induced autophagy and inhibited Aurora A activity when combined with FDA-approved anticancer drugs [37]. Liu group reported that Aurora A kinase suppresses metabolic stress-induced autophagic cell death by activating mTOR signaling in breast cancer cells, providing a novel insight into the cytoprotective role of Aurora A against metabolic stress, which might help to develop alternative cell death avenues for breast cancer therapy [38]. Furthermore, Aurora A inhibitor Alisertib induces autophagy in various human cancer cells [39,40,41,42]. However, to the best of our knowledge, the role of Aurora B in autophagy regulation has not been reported.

Overexpression of Aurora A and B has been observed in several tumor types and has been linked with a poor prognosis for cancer patients. Noteworthy are clinical trials in which several Aurora kinases inhibitors have been tested (phase I and II trials) [43]. Hence, further illustration of the detailed mechanism of Aurora kinases in mitosis and autophagy regulation will not only be helpful to understand the tumorigenesis of cancers that are sensitive to Aurora kinases inhibition, but also guide the future combinational chemotherapy for Aurora A overexpression-related cancers.

### 2.3. Polo-Like Kinases

PLK, also known as polo-like kinase, is a ubiquitously expressed serine/threonine protein kinase. The PLK family (PLK1 to 5) members all contain a polo-box domain that regulates their kinase activity and subcellular localization [44,45]. PLK1 is the most investigated PLK protein and is an early trigger for G2/M transition. Long-term PLK1 inhibition by its inhibitors, such as BI2536, arrests cells in prometaphase and thus PLK1 inhibitors are investigated as antimitotic agents for cancer treatment considering its high expression in tumors [45,46].

Although many studies suggest that PLK1 is an autophagy regulator because the PLK1 inhibitor affects autophagy, whether PLK1 induces or inhibits autophagy still remains controversial. While some groups declare that inhibiting PLK1 induces autophagy to different extents, other groups demonstrated autophagy attenuation. For example, in 2015, Valianou et al. showed that BI2536 moderately inhibits autophagy in TSC1/2 (Tuberous sclerosis proteins 1/2)-deficient lymphangioleiomyomatosis patient derived cells, which suggested that PLK1 might be an autophagy inducer [47]. Recently, however, Ruf et al. suggested that PLK1 induces autophagy more obviously by mTORC1 inhibition, which was determined by the reduction in LC3-II accumulation and autolysosomes number induced by PLK1 inhibition [46]. As TSC1/2 are negative regulators of mTORC1, loss of TSC1 or TSC2 leads to mTORC1 hyperactivation. Therefore, mTORC1 could not be further activated by PLK1 inhibition in a TSC1/TSC2-deficient background in a study by Valianou et al., which may explain why only a moderate effect of BI2536 on autophagy was observed. In contrast, another study by Deeraksa et al. on LNCaP cells reports that long-term treatment with BI2536 for 5 d leads to mitotic arrest and necroptosis, correlating with cell death related autophagy activation, which indicates an autophagy inhibition function of PLK1 [48]. It should be noted that in Ruf’s report, autophagy was decreased in HeLa cells upon a 38h consecutive aphidicolin-nocodazole block-induced mitotic block. Moreover, it has been shown that BUB1 inhibited autophagy in breast cancer cell line MCF-7 but not in immortalized MCF-10A breast epithelial cells. Thus, autophagic activity during mitosis may vary depending on the length of cell cycle arrest and cell types, which adds variations to this paradox.

### 2.4. NimA-Related Protein Kinase 4 (NEK4 Kinase)

NEK4, also known as Never in mitosis gene A-related kinase 4 or NimA-related protein kinase 4, is a serine/threonine protein kinase which belongs to the NIMA family including NEK1–NEK7 seven members in mammalian genome [49]. NEK4 was required for normal entry into proliferative arrest after a limited number of cell divisions, also called replicative senescence, and for normal cell cycle arrest in response to double-stranded DNA damage [50]. Recently, a siRNA screening identified that NEK4 suppresses autophagosome formation downstream or independent of mTORC1 [32]. Another study declared that NEK4 inhibition potentiates TRAIL-induced cell death in TRAIL (tumor necrosis factor-related apoptosis inducing ligand)-resistant cancer cells. It was found that TRAIL-induced cell death in NEK4 knockdown cells was completely blocked by a pan caspase inhibitor, Z-VAD, but not by the necrosis inhibitor, necrostatin-1, or by the autophagy inhibitor, Bafilomycin A1 [51]. It suggests that NEK4 inhibition mediated autophagy induction does not contribute to these types of cell death.

### 2.5. Budding Uninhibited by Benzimidazoles 1 (BUB1) Kinase

Mitotic checkpoint BUB1 (budding uninhibited by benzimidazoles 1) is a serine/threonine protein kinase. It includes a conserved N-terminal region, a central non-conserved region and a C-terminal serine/threonine kinase domain [52]. The protein level and the kinase activity of BUB1 are regulated during the cell cycle, during which they peak in mitosis and are down-regulated in the G1/S phase [53]. During prophase it localizes to the outer kinetochore, a process generally implicated in correcting mitotic timing and checkpoint response to spindle damage. BUB1 possesses versatile and distinct functions during the cell cycle, mainly in the spindle assembly checkpoint (SAC) and chromosome alignment during metaphase [54].

Recently, the Kitagawa group discovered that BUB1 mediated caspase-independent mitotic death (CIMD), possibly through autophagy [55]. Moreover, a comprehensive siRNA screen indicated the involvement of BUB1 in autophagy regulation. Interestingly, as mentioned above, BUB1 inhibited autophagy in breast cancer cell line MCF-7 rather than immortalized MCF-10A breast epithelial cells, which indicated the anti-cancer potential of BUB1 modulation [32]. Consistently, siRNA-mediated knockdown of the SAC component Mad2 or BUB1 also led to an increase in LC3-II level [56].

Although both BUB1 and BUBR1 (budding uninhibited by benzimidazole-related 1) are central components of the SAC, currently only BUB1 rather than BUBR1 was reported to participate in autophagy regulation. Despite their amino acid sequence conservation and similar domain organization, BUB1 and BUBR1 perform different functions in the SAC [54]. If BUBR1 plays different roles in autophagy other than BUB1, it would suggest that autophagy might be associated with differentially finely-tuned SAC. Therefore, further study is needed to investigate BUBR1 function in autophagy regulation.

### 2.6. Mitogen-Activated Protein Kinases (MAPKs)

Cells recognize and respond to extracellular stimuli to activate the mitogen-activated protein kinases (MAPKs), which are implicated in cell cycle and autophagy regulation. To date, five distinct groups of MAPKs have been characterized in mammals: extracellular signal-regulated kinases (ERKs) 1 and 2 (ERK1/2), c-Jun amino-terminal kinases (JNKs) 1, 2 and 3, p38 isoforms α, β, γ and δ, ERKs 3 and 4, and ERK5 [57,58]. The most extensively studied groups of MAPKs to date are the p38, ERK1/2 and JNKs kinases. In general, MAPKs can be activated by a wide variety of stimuli. Specifically, the p38 and JNK kinases are more responsive to stress stimuli ranging from osmotic shock and ionizing radiation to cytokine stimulation, while ERK1 and ERK2 are preferentially activated in response to growth factors and phorbol esters [59].

MAPK plays an important role in the regulation of apoptosis, cell cycle arrest, growth inhibition and differentiation [60]. Among those, the p38 MAPK pathway in the regulation of mitotic entry has been well established in non-stress conditions and in response to various genotoxic stresses such as DNA damage [61,62,63]. p38 γ MAPK was identified as a modulator of mitotic progression and mitotic cell death, which set up a new link between the p38 MAPK pathway and the mitotic signaling network [64]. Moreover, p38 MAPK functions in the regulation of autophagy. MAPK was not only a positive regulator in oridonin, E Platinum and the heme oxygenase-1 inhibitor ZnPPIX-induced autophagy but also a negative regulator in TNFα and Triterpenes induced autophagy [65,66,67,68,69]. Mechanistically, some molecular targets have been implicated in p38 MAPK-induced up- or down-regulation of autophagy, which includes phosphorylation of ATG5, competition with mAtg9 for p38IP binding, as well as phosphorylation of glycogen synthase kinase 3β (GSK3β) [70,71,72]. Considering the roles of p38 MAPK in mitotic entry and autophagy, it will be interesting to unravel the internal link of both processes by identifying the common interacting partner.

Erk1/2 are implicated in cell cycle regulation such as G1/S transition and G2/M transition. Importantly, a strict control of the kinetics and strength of Erk activity is critical for G2/M transition. Mechanistically, Erk participates in the nuclear translocation of cyclin B1, blocking phosphorylation of Cdc2 by Myt1 [73]. Recently, the RAS-regulated Erk1/2 pathway was also indicated in the control of mitotic spindle angle which determines lung tube shape [74]. Besides their roles in mitosis, Erk1/2 were involved in autophagy regulation. For example, the Erk1/2 pathway is implicated in the deregulation of autophagy, lipid metabolism related autophagy, autophagy mediated survival program and autophagic dynamics in cancer cells [75,76,77,78].

JNK was suggested to control the onset of mitosis and mitotic spindle regulation [79,80,81]. JNK degradation by APC/C-Cdh1 occurred during exit from mitosis and non-degradable JNK induces prometaphase-like arrest and aberrant mitotic spindle dynamics [82]. Moreover, JNK-mediated Cdc25C phosphorylation regulates cell cycle entry and G2/M DNA damage checkpoint [83]. In response to stress signals, JNK also contributes to autophagic induction. JNK signaling pathways participate in various small molecule compounds induced autophagy, autophagy dependent chemotherapeutics resistance and HBx-induced autophagy [84,85,86,87]. Mechanistically, JNK activation modulates autophagy through two distinct mechanisms. On the one hand, it promotes Bcl-2/Bcl-xL phosphorylation, resulting in the dissociation of the Beclin-1 with Bcl-2/Bcl-xL. The liberation of Beclin-1, an essential autophagy modulator, thereby stimulates autophagy [88,89,90,91,92]. On the other hand, JNK leads to the upregulation of damage-regulated autophagy modulator (DRAM). DRAM can stimulate autophagosomes accumulation by regulating autophagosome-lysosome fusion to generate autolysosomes [93,94,95].

Furthermore, ERK8/MAPK15 was discovered as a new member of the MAPK family by Rosner and colleagues in 2002. Although ERK8 is closely related to ERK7, it was found to be activated in a Src-dependent manner, which is different from the ERK7 activation signaling pathway [96]. Interestingly, ERK8 was later described in the regulation of autophagy by Chiariello and colleagues in 2012. By interacting with ATG8-like proteins (MAP1LC3B, GABARAP and GABARAPL1), ERK8 localizes to autophagic compartments and stimulates autophagy [97].

## 3. Kinases Originally Involved in Autophagy Regulation Are Shown to Play Additional Roles in Mitosis

### 3.1. Mechanistic Target of Rapamycin Complex 1 (mTORC1)

mTOR (known as Mechanistic Target of Rapamycin) belongs to the serine/threonine protein kinase, which is involved in cell growth and cell proliferation by way of forming two distinct complexes, mTORC1 and mTORC2 [98]. As a multifunctional protein kinase, mTOR plays vital roles in autophagy regulation as well. In fact, it is one of the most well studied kinase regulators of autophagy. mTORC1 controls autophagy induction negatively in response to nutrient starvation, stress and reduced growth factor signaling. mTORC2 regulates autophagy via Akt–FoxO3 in skeletal muscle cells in response to a fasting condition [99]. Predominantly, mTORC1 controls autophagy through ULK1–ATG13–FIP200 pathway in which ULK1–Ser757 and ATG13–Ser258 were phosphorylated by mTORC1 in nutrient rich conditions [100,101]. However, besides its well-known function in autophagy, recent data suggested that mTOR and its co-factors were regulated in mitosis. For example, phosphorylation of mTOR–Ser2481 couples with chromosome condensation and segregation during mitosis [102,103]; mTORC1 controls spindle function during mitosis and meiosis, which is supported by co-localization of Raptor (Regulatory-associated protein of mTOR, the core component of mTORC1) and mTOR on the spindle [104]; mitotic raptor was hyperphosphorylated in mitosis, which promotes mTORC1 activity, G2/M cell cycle progression, and internal ribosome entry site-mediated mRNA translation [105,106]. Moreover, 4E-BP1—one of the major substrates of mTOR—was also hyperphosphorylated in mitosis to contribute to cap-dependent translation [107]. However, in the mitotic context, the precise role of mTORC1 in autophagy regulation still needs to be determined, which is possibly helpful for cancer therapy by targeting both mitotic progression and autophagy regulation simultaneously through mTOR modulation.

### 3.2. AMP Activated Protein Kinase (AMPK)

AMPK (AMP activated protein kinase) is an energy sensor which is activated when intracellular ATP levels are low. AMPK is a conserved heterotrimeric serine/threonine protein kinase composed of a catalytic α subunit, a scaffolding β subunit, and a regulatory γ subunit. Of those, AMPKα subunit, existing as either the α1 or α2 isoform, is the core component for AMPK activity. Previous data have shown that AMPK coordinates with mTOR for nutrient and energy balance in cells to control autophagy [108,109]. Several recent papers have addressed a new mechanism for the control of mammalian autophagy by AMPK, which interacts with phosphorylates and activates ULK1 [100,110,111,112].

Other than its role in autophagy, AMPK was also implicated in mitosis. For example, phosphorylation of AMPKα T172 residue was negligible in interphase but reached a maximum in mitosis, which re-localized phosphorylated AMPKα T172 to chromosomal passenger [113,114]. Functionally, AMPKα regulates mitotic spindle orientation through phosphorylation of myosin regulatory light chain [115]. Moreover, Polo-like kinase 1 regulates activation of AMPKα at the mitotic apparatus and the LKB1 tumor suppressor controls spindle orientation and localization of activated AMPKα in mitotic epithelial cells [116]. Importantly, a chemical genetic screen for AMPKα2 substrates uncovers a network of proteins involved in mitosis [117], which further confirmed the key roles of AMPK in mitosis. Given the fact that AMPK is an energy sensor regulating autophagy and mitosis is a high energy demand process, both autophagy and mitosis will be tightly connected by AMPK. Thereby, it was necessary to dissect the detailed mechanisms of AMPK in mitotic autophagy. In addition, the functions of the β and γ subunits of AMPK in mitosis still remain unclear.

### 3.3. Phosphoinositide-3 Kinase (PI3K) and Protein Kinase B (AKT)

PI3K (Phosphoinositide-3 kinase) is a family of enzymes that are capable of phosphorylating phosphatidylinositol (PtdIns) at 3′-hydroxyl group on the inositol ring. PI3K comprises of Class I, Class II, Class III groups [118]. AKT, also known as PKB (protein kinase B), is a serine/threonine kinase originally identified as a cellular homolog of the viral oncogene Akt8. Class I PI3K functions primarily through the PI3K/AKT pathway, which is activated by several membrane receptors such as receptor tyrosine kinases (RTKs) and G protein-coupled receptors (GPCRs). Activated Class I PI3K catalyzes its substrate, PtdIns (4,5) P2, to produce PtdIns (3,4,5) P3, which recruits AKT to plasma membrane, where it is phosphorylated on T308 and S473 for full activation. Activated AKT finally leads to the activation of mTORC1 via TSC and Rheb, which inhibits autophagy in an mTOR-dependent manner [119,120]. Class III PI3K, also known as VPS34, induces autophagy through the generation of PtdIns (3) P to recruit other autophagic proteins to form VPS34 complex [121,122,123].

In addition to autophagy, the PI3K/AKT pathway was also shown to function in mitosis. In synchronized cell lines, AKT is active during mitosis and the inhibition of the PI3K/AKT pathway promotes a delay in S phase exit and G2/M transition due to a decrease in CDK1 activity [124,125,126]. In addition, AKT inhibition regulates Aurora A kinase expression, which interferes with centrosome separation, mitotic progression and bipolar spindle formation [127]. Moreover, functional PI3K associates with CDK, small GTPases such as Rab11 and Rho family to contribute to mitotic entry, metaphase progression and spindle orientation while PI3K signaling was attenuated during anaphase and telophase [128].

Similarly, the class III PI3K VPS34 also functions in cytokinesis, which is the final step of mitosis. The PI3K class III sub-complex containing VPS15, VPS34, Beclin-1, UVRAG and BIF-1 regulates cytokinesis via its product PtdIns (3) P and some subunits such as VPS34 or Beclin-1 [129,130]. During cytokinesis, PtdIns (3) P-positive endosomes localize at the midbody and the inhibition of PtdIns (3) P synthesis by PI3K inhibitors induces cleavage furrow regression and cytokinesis arrest [131,132]. Moreover, Ric-8A, a guanine nucleotide exchange factor for Gα_i_, Gα_q_, and Gα_12/13_, contributes to cytokinesis abscission by controlling VPS34 kinase activity [133]. Although VPS34 complex functions in autophagy and cytokinesis, whether its autophagic activity is involved in cytokinesis remains elusive.

## 4. Conclusions

In conclusion, there is increasing evidence showing that multiple kinases could regulate both cellular processes (Table 1). Given the fact that autophagy is differentially regulated throughout the cell cycle, the dual roles of these kinases could provide the basis for further experimental and theoretical work about the interplay of the two dynamic processes.

## 5. Perspectives

Although kinases are critical regulators of essentially all cellular processes, so far only a few kinases are found to be involved in autophagy and even fewer were shown to participate in both autophagy and mitosis [8,9,134]. Mitosis is an open process with chromosomes, dispersed Golgi, and nuclear contents all exposed in cytoplasm, which is vulnerable to autophagic nonspecific bulk degradation. The underlying mechanism for mitotic kinases associated autophagy regulation will help us acquire a more complete understanding about how cells protect the integrity of their mitotic apparatus and ensure accurate mitosis. Moreover, the findings about kinases with dual roles in autophagy and mitosis will be implicated in relevant clinical diseases.

Recently, ULK3, a member of Unc-51-like kinase, was implicated in the regulation of cytokinetic abscission by phosphorylating ESCRT-III proteins other than its previously known function in autophagy [135,136]. However, whether ULK1/2—the known key regulators of autophagy—are involved in mitosis is still unknown. In fact, generally speaking, whether other members of the same kinase family have similar autophagy regulation functions still remains elusive. For example, do CDK2 and CDK4, Aurora B, PLK2 and BUBR1 all participate in autophagy regulation like their family members CDK1, Aurora A, PLK1 and BUB1? At the same time, identification of upstream kinases or downstream executors of the identified kinases will be necessary for uncovering the unconventional roles of such kinases. More importantly, whether these kinases use the same set of downstream players/substrates to regulate autophagy and mitosis, and how their functions are differentially controlled in the two distinct cellular events still need further investigations. Furthermore, up to now only very few signaling pathways such as PI3K/AKT/mTOR were implicated in stress-induced autophagy—whether other mitotic kinases pathways function in autophagy also needs to be investigated.

Considering such kinases’ dual roles in mitosis and autophagy, whether mutual regulation occurs between both processes controlled by the same kinase remains elusive. For example, does autophagy activity regulated by CDK1 affect its function in G2/M transition? Is mTORC1 localization and function in mitosis regulated by its roles in autophagy? Thus, illustration of common regulators and their intertwined regulation network in both mitosis and autophagy will strengthen the internal link between these two highly dynamic cellular processes. More speculatively, it could shed light on combinatorial therapy for intractable diseases such as cancer, in which both mitosis and autophagy play irreplaceable roles.

## Figures and Tables

**Table 1:** Kinases involved in mitosis and autophagy regulation.

Kinases	Originally Found to Function in Mitosis or Autophagy	Later Evidences for Their Function in the Other Process
Aurora A	Mitotic spindle formation and centrosome separation [35,36]	Autophagy inhibition [37,38,39,40,41,42]
CDKs	Cell cycle regulation (primarily in mitosis by CDK1 and CDK11) [5,22]	Autophagy regulation [12,13,23]
PLK1	G2/M transition [45,46]	Autophagy induction [46,47].
NEK-4	Cell cycle arrest [50]	Autophagy inhibition [32]
BUB1	Spindle assembly checkpoint and chromosome alignment [53,54]	Autophagy inhibition [32,55]
MAPKs	P38: mitotic entry [64], Erk1/2: G2/M transition [73,74], JNK: onset of mitosis and mitotic spindle regulation [79,80,81]	P38: dual roles in autophagy regulation [65,66,67,68,69], Erk1/2: autophagy regulation [75,76,77,78], JNK: autophagy induction [84,85,86,87]
mTORC1	Starvation induced autophagy [100,101]	Mitotic progression [102,103,104,105,106,107]
AMPK	Energy associated autophagy [110,111,112]	Mitotic spindle orientation [113,114,115,116,117]
PI3K/AKT	Induce or inhibit autophagy [119,120,121,122,123]	G2/Mitosis transition and cytokinesis [124,125,126,127,128,129,130,131,132]
